# Commentary: Snap-N-Send: A Valid and Reliable Method for Assessing the Energy Intake of Elite Adolescent Athletes

**DOI:** 10.3389/fnut.2017.00047

**Published:** 2017-09-29

**Authors:** Nessan Costello, Jim McKenna, Kevin Deighton, Ben Jones

**Affiliations:** ^1^Carnegie Faculty, Institute for Sport, Physical Activity and Leisure, Leeds Beckett University, Leeds, United Kingdom; ^2^Leeds Rhinos RLFC, Leeds, United Kingdom; ^3^Yorkshire Carnegie, Leeds, United Kingdom; ^4^The Rugby Football League, Leeds, United Kingdom

**Keywords:** behavior change, behavior change wheel, dietary assessment, elite athletes, validity and reliability

Diet is an ever-changing, poorly characterized, and multifaceted phenomenon. Consequently, traditional dietary assessment methods demonstrate considerable random intra- and interindividual day-to-day variation and systematic over- or underreporting bias [errors of reliability and validity; (1, 2)] across populations (3). Expressed practically, true assessments of energy intake are misrepresented by hundreds of calories per day (4), erroneously informing medical conclusions (5), media claims (6, 7), and national dietary guidelines (8). Ultimately, the enormous potential of nutrition research to drive national health, patient welfare, and public service (9) urgently necessitates and ethically obligates the valid assessment of diet within all dietetic output.

Technological advances have enabled development of a new generation of electronic dietary intake assessments (e-DIA), operating across several platforms [internet, sensor, mobile; (10)]. e-DIA support previously unachievable assessment ideologies, such as ecological momentary assessment [EMA; (11)], allowing for the rapid collection, management, and storage of dietary information as it occurs in the habitual environment of participants (12). Nonetheless, many objective e-DIA remain limited by their poor accessibility (i.e., expense) and inability to translate into actual dietary or energy intakes (10). Such methods require further development (10) and robust validation (13) before their measurement sensitivity can be confirmed. Alternatively, self-reported e-DIA are highly accessible, providing enhanced validity over traditional approaches (14). Nevertheless, such methods are still subject to the considerable measurement error that confounds traditional self-report dietary assessment; evidently, a new and improved approach is required.

In light of these limitations, we propose a novel behavioral approach within the valid assessment of diet. This approach recasts self-report dietary assessment as both potentially valid and reliable (9), allowing for possibly unique distinction between methodological and behavioral (15) measurement error. Methodological measurement error is inherent within the innate design of a dietary assessment tool. For example, the finite food items listed by a food frequency questionnaire (FFQ), the recall bias within memory-based assessment methods [M-BMs; (6, 7)], or “estimation” involved within an estimated food diary (16). Such dietary assessment tools cannot be absent of methodological measurement error even when completed correctly by a behaviorally adhered participant. Alternatively, behavioral measurement error emerges from poor participant “capability” and/or “motivation” (17) to complete any dietary assessment in exact accordance with the method design, for the entire recording period. For example, poor literacy skills might affect the “capability” of an individual to comprehend the questions within a FFQ, whereas, poor “motivation” might result in the completion of a weighed food diary *via* estimation, rather than actually weighing dietary consumption as designed (16). It is now clear that methodological measurement error is the sole focus of current dietary assessment critique (4), research (10), and design innovation (18). However, whereas methodological error can be attenuated by appropriate dietary assessment tool selection (19), behavioral error requires unique, and often over-looked, addressment.

Leading behavior change science, as summarized by the Behavior Change Wheel [BCW; (20)], can be used to define population-specific behavioral barriers to the accurate recording of diet; attenuating, if not entirely eradicating, behavioral measurement error. The Capability, Opportunity, Motivation—Behavior model (COM-B) outlines how to effectively change the desired behavior, through nine intervention functions and seven categories of policy. The systematic, theoretical, and applied nature of the BCW, summarized into eight easy-to-understand implementation steps, makes it an outstanding and pragmatic choice to achieve valid dietary assessment. In this regard, we have recently validated a behavioral approach within a challenging population of elite adolescent athletes. Forty-seven behavior change techniques were identified and delivered across six intervention domains and five categories of policy to overdetermine correct and habitual adherence to real-time protocols (EMA) utilizing an innovative method [“Snap-N-Send”; (21)]. Findings strongly evidence the importance of deploying comprehensive behavior change science alongside innovative technology to secure improved adherence to real-time protocols and more valid self-reported dietary assessment.

Subsequently, a behavioral approach can be used to prevent complex biases, often accepted as innate (15) shortcomings within self-report dietary research. By ensuring, rather than assuming, that participants are both behaviorally “capable” and “motivated” to record what they consume, social desirability, and reactivity bias can be attenuated, if not completely prevented. Furthermore, a behavioral approach which confirms high participant adherence to real-time assessment protocols (EMA) can also attenuate, if not theoretically prevent, the extensive memory-based bias apparent within epidemiological research (6, 7). Additionally, increased participant “capability” and/or “motivation” most likely explains why many innovative e-DIA now report improved validity and reliability (10, 21) over traditional, often laborious self-report methods (16). Ultimately, further successful attenuation of measurement error within dietary assessment hinges upon effective deployment of primary behavior change science into the design and delivery of innovative or existing dietary intake assessment.

To conclude, diet is the product of dynamic behavioral and environmental exposure, which presents unique challenges for methodological design and valid assessment. Left unattended, this dynamism produces substantial methodological and behavioral measurement error, which undermines confidence in assessment outcomes. Although there have been improvements in the execution of dietary assessments (10), these have been insufficient to offset calls to abandon self-report assessment altogether (4). New eclectic models of behavior change (e.g., COM-B) are now available to guide the design of bespoke instruments that address behaviors that impede sustained accurate dietary reporting. This new scientific domain represents an original and effective approach to reduce and even prevent dietary assessment measurement error. Using this approach effectively, signals a paradigm shift in expectations for instrument design and implementation within the valid assessment of diet.

## Author Contributions

All authors (NC, JM, KD, and BJ) were authors on the original publication which has been commented on (Snap-N-Send: A Valid and Reliable Method for Assessing the Energy Intake of Elite Adolescent Athletes). All authors have contributed substantially to this general commentary, in regards to the text and concept behind the argument.

## Conflict of Interest Statement

The authors declare that the research was conducted in the absence of any commercial or financial relationships that could be construed as a potential conflict of interest.
